# Administration of Atropine Eye Drops Sublingually for Clozapine-Induced Sialorrhea in Bipolar Disorder

**DOI:** 10.1192/bjo.2024.663

**Published:** 2024-08-01

**Authors:** Shaurya Garg, Amit Kumar, Omar Afroz, Preethy Kathiresan, Raman Deep

**Affiliations:** All India Institute of Medical Sciences Delhi, New Delhi, India

## Abstract

**Aims:**

The aim of this study is to explore the off-label use of atropine, administered sublingually, for the management of clozapine-induced sialorrhea in a patient who showed inadequate response to commonly used agents. The investigation stems from a clinical scenario where traditional approaches failed, prompting an exploration of alternative and cost-effective options to alleviate sialorrhea associated with clozapine therapy in a patient of lower socio-economic status.

**Methods:**

Mr. A, a 29-year-old with bipolar affective disorder, experienced persistent sialorrhea during clozapine treatment, resistant to trials with trihexyphenidyl and glycopyrrolate. Following a brief discontinuation of clozapine, the patient relapsed into a manic episode, leading to hospitalization. Despite the re-initiation of clozapine, sialorrhea reoccurred. Various doses and combinations of trihexyphenidyl and glycopyrrolate were ineffective, with affordability issues limiting the latter. As sialorrhea persisted, clozapine dose reduction was necessary. Attempts with different antipsychotics were made, and valproate sodium was increased, but sialorrhea remained problematic.

Given the patient's unique case and previous medication failures, an off-label use of atropine via a sublingual route was done after obtaining informed consent. Quantitative measurement of sialorrhea was conducted using a sialometry machine. The patient underwent a trial with sublingual atropine drops, and the salivary rate significantly decreased, indicating a potential efficacy in managing clozapine-induced sialorrhea.

**Results:**

The discussion encompasses the challenges faced in managing clozapine-induced sialorrhea in the presented case. Traditional agents, including glycopyrrolate and trihexyphenidyl, proved ineffective or were hindered by affordability issues. The subsequent reduction of clozapine dose compromised overall treatment efficacy. The introduction of atropine eye drops via sublingual administration emerged as a novel approach, demonstrating a reduction in salivary rate without notable adverse effects except elevated heart rate 2 hours after administration of atropine. The unique pharmacological properties of atropine, despite being an off-label use, provided a potential avenue for addressing persistent sialorrhea.

**Conclusion:**

In conclusion, the off-label use of atropine via the sublingual route showed promise in alleviating clozapine-induced sialorrhea in this particular case. Despite demonstrable efficacy in pre and post-sialometry, the clinical challenges and practical considerations associated with atropine's use in this context raise concerns. The case underscores the need for alternative strategies in managing medication-induced side effects, especially when standard interventions fail. Further research is warranted to explore the broader applicability and safety of this approach in a larger cohort.

